# A Review of Patient-Reported Outcomes and Clinical Outcomes in Acute and Chronic Myeloid and Lymphoid Leukemias

**DOI:** 10.3390/hematolrep18010015

**Published:** 2026-02-06

**Authors:** Bryan Chan, Eesha Balar, Seiichi Villalona, Judith Karp, Allison Leahy, Catherine Lai

**Affiliations:** 1 Department of Hematology and Medical Oncology, Harbor-UCLA Medical Center, Torrance, CA 90502, USA; 2 Perelman Center for Advanced Medicine, University of Pennsylvania, Philadelphia, PA 19104, USA; 3 Johns Hopkins Sidney Kimmel Comprehensive Cancer Center, Baltimore, MD 21231, USA; 4 Center for Childhood Cancer Research, Children’s Hospital of Philadelphia, Philadelphia, PA 19104, USA

**Keywords:** patient-reported outcome (PRO), patient-reported outcome measurement (PROM), leukemia, health-related quality of life (HRQoL), clinical outcomes

## Abstract

**Introduction:** This review specifically focuses on interventional clinical trials in leukemias and myelodysplastic syndromes (MDS), summarizing how patient-reported outcome measures (PROMs) have been implemented to evaluate treatment effects rather than to directly influence clinical outcomes. **Objective:** Clinical outcomes of interest typically include response rates, disease-free survival (DFS), and overall survival (OS). Patient-reported outcome measures (PROMs) are standardized questionnaires that collect information regarding health outcomes directly from the patient and are used to evaluate new treatments and healthcare quality. In addition, the use of PROMs in cancer care has been shown to improve patient-provider communication and patient satisfaction. **Material and Methods:** This is a qualitative, narrative synthesis and review structured around PROMs focused on six critical themes: symptoms/symptom burden, physical, emotional, social/role, and functional status, and global health status measurement. **Results:** PROMs that are assessed in oncologic research include the EORTC QLQ-C30, FACT-Leu, QLQ-CLL16, and EQ-5D. PROs are associated with clinical outcomes such as DFS and OS, and the FACT-Leu scales, HRQOL and physical functioning scores were independent prognosticators of OS in patients with AML. **Conclusions:** Through our review, notable trends were identified that further highlight the importance of greater incorporation of PRO measures in future clinical trials, particularly in the understudied realm of hematologic malignancies, in order to better delineate the link between survival and HRQOL.

## 1. Introduction

Patient-reported outcome measures (PROMs) are standardized questionnaires that collect information regarding health outcomes directly from the patient and are used in healthcare research to evaluate new treatments, supportive care interventions, and healthcare quality. The FDA defines patient-reported outcomes as “a measurement based on a report that comes directly from the patient (i.e., study subject) about the status of a patient’s health condition without amendment or interpretation of the patient’s response by a clinician or anyone else” [1,2,3].

PROMs have undergone psychometric validation and standardization to include pertinent questions regarding how symptoms and functional status can reliably assess changes over time [4,5]. Broadly, PROMs fall into two main categories: symptomatology and functional status (either physical, emotional, social/role), which are often associated with the broader concept of health-related quality of life (HRQOL). As one example, the EQ-5D (EuroQol-5D) measures health concepts that are relevant to a wide range of patient groups, and is multi-dimensional in its construct. This instrument, developed by the EuroQol Group, asks questions regarding the patient’s health, mobility, self-care, usual activities, pain symptoms and anxiety/depression [5]. Condition-specific PROMs capture elements of health relevant to a particular patient group or condition, such as those devised by the European Organization for Research and Treatment of Cancer (EORTC), which developed a widely used PROM that assesses the quality of life of patients with cancer, the EORTC Core Questionnaire QLQ-30 [5,6]. This 30-item PROM, developed over a decade of collaborative research, assesses diverse determinants of quality of life of cancer patients and similar rigorously developed instruments exist from a variety of sources, including the National Institute of Health (NIH) funded Patient-Reported Outcomes Measurement Information System (PROMIS).

To holistically address the assessment of symptom burden and HRQoL using PROs in relation to trial outcomes for acute and chronic myeloid and lymphoid leukemias, we conducted a narrative, qualitative literature review to synthesize and compare diverse elements of PROM (global health status, symptomology, emotional function, physical function, social/role function) on evaluation of treatment impact and patient well-being, rather than direct determination of clinical outcomes. The purpose of this review is to (a) summarize PROM that have been used in clinical trials involving leukemias and myelodysplastic syndromes (MDS), and (b) synthesize how PRO have been applied to assess treatment effects and patient well-being in these populations. Importantly, this review does not posit a causal effect of PROM on clinical outcomes. Administering questionnaires or collecting PRO data does not alter survival or treatment efficacy per se. Rather, PRO data collected in clinical trials provide insights into symptom burden and HRQoL, which inform understanding of treatment tolerability and patient-centered benefit, including how the integration of patient-reported outcomes can inform clinical decision-making, enhance patient-centered care, and complement traditional survival-based endpoints in leukemia and MDS trials.

## 2. Methods

**Figure 1:**
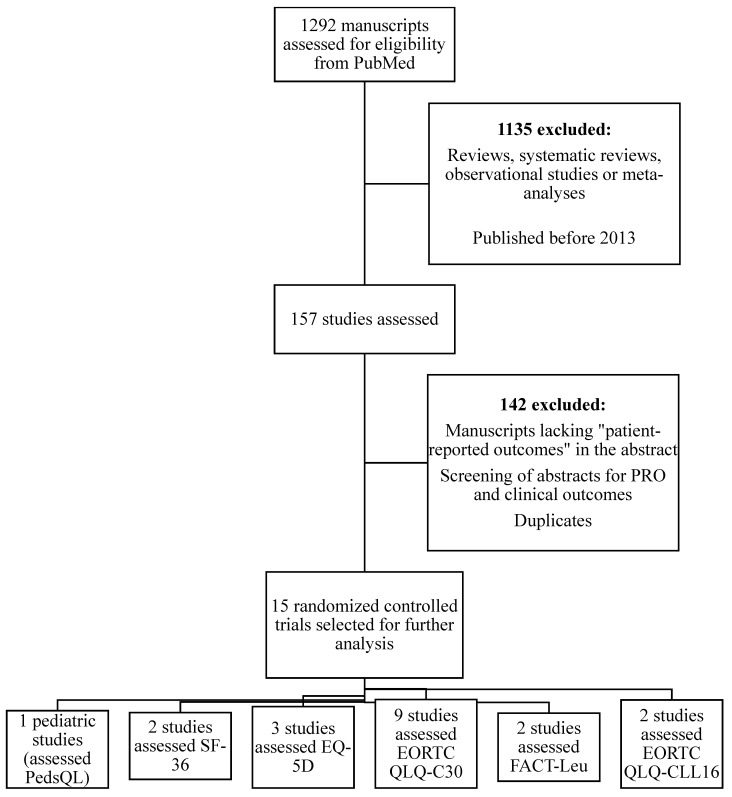
Selection process of literature review in myeloid diseases and leukemias with patient-reported outcome instruments.

MEDLINE and the Cochrane Library were searched for studies published between 2013 and 2025; this search was conducted in 2025. The search strategy initially consisted of terms or keywords in MEDLINE such as “patient-reported outcomes,” which were used pragmatically to narrow the initial search given the high volume of available literature; eligibility was based a priori on assessment of outcomes directly reported by patients via named instruments or constructs (e.g., health-related quality of life), regardless of whether the terms “PRO” or “PROM” were explicitly stated in the title or abstract and “AML”, “CML”, “ALL”, “CLL”, or “MDS”. Studies were considered eligible for inclusion if they enrolled patients with myelodysplasia (MDS) and/or acute or chronic leukemias (AML, CML, ALL, or CLL) that included both PROM and measures of survival such as OS, DFS, event-free survival (EFS) and progression-free survival (PFS). Studies were eligible if they enrolled patients with myelodysplastic syndromes (MDS) and/or acute or chronic leukemias (AML, CML, ALL, or CLL) and used validated PROM within interventional clinical trials. Observational studies were excluded to maintain methodological consistency, although this limits exploration of PRO–survival associations typically derived from observational data. Studies were also excluded for the following criteria: (1) written in a language other than English; (2) not peer-reviewed; and/or (3) conference reports or editorial papers. In addition, observational studies were excluded given potential heterogeneity in outcomes and methodological limitations. A total of 1292 studies were identified for initial review using the search criteria noted, which were then carefully narrowed to 157 articles based on the above criteria and objectives of the review using title and abstract preliminary screening. Ultimately, 15 articles met the inclusion criteria, with articles that focused on the importance of PROM and measures of survival (often through interventions) being selected to better guide our discussion (Table 1). Search terms consisted of Medical Subject Headings (MeSH) and free-text words. In addition to the primary search method identified above, additional references were obtained by hand-searching reference lists of included studies and systematic reviews (backwards selection) and identifying studies that cited the original included studies (forward selection) using both Cochrane Library and Google Scholar [Figure 1]. Duplicate studies were removed [Appendix A]. A qualitative (rather than systematic) synthesis structured around PROM focused on six critical themes: symptoms/symptom burden, physical, emotional, social/role, and functional status, and global health status measurement. In addition, our purpose was to (a) summarize PRO instruments that have been used in clinical trials of patients with leukemias and MDS, and (b) to synthesize findings regarding how PROs have been applied in these trials. This work was conducted as a narrative (qualitative) literature review. We utilized established approaches for narrative synthesis, including Synthesis without Meta-analysis (SWiM) principles and the IMRAD (Introduction, Methods, Results, and Discussion) format. Accordingly, the EQUATOR network’s SANRA (Scale for the Assessment of Narrative Review Articles) guideline was justly applied [7].

## 3. Results

### 3.1. Patient-Reported Outcome Measure (PROM)

Given the poor survival outcomes for the majority of AML and MDS patients, evaluating HRQOL can facilitate treatment decisions. Patient-reported outcomes were assessed at heterogeneous timepoints across trials, including baseline (pre-treatment), on-treatment, and post-treatment or survivorship phases; these assessments reflect distinct clinical constructs, ranging from pre-existing symptom burden to treatment-related changes and longer-term recovery or late effects. The influence of the recently developed approaches of less intensive therapies, targeted therapies, and immunotherapies has limited data. New disease-specific instruments such as the Quality of Life in Myelodysplasia Scale (QUALMS), and the Quality of Life for Acute Myeloid Leukemia (AML-QOL15) are in the validation stages and may be influential in the future management landscape [19]. In addition, the integration of PROMs in routine clinical care has also been shown to impact clinical outcomes in ways such as increased patient satisfaction, which may influence the overall quality of care in patients with chronic, severe ailments [20]. PROMs that are often used to assess leukemic patients in clinical trials include the EORTC QLQ-C30, FACT-Leu, QLQ-CLL16, and EQ-5D; for studies involving pediatric patients, one relatively common measure is the PedsQL [9,21]. A review of the studies representing diverse instruments and leukemias in clinical trials in the target population is depicted in Table 1 (a more detailed overview to be found in the chart), with each study showing the results of various interventions on both PRO and clinical outcomes (primarily for adults, with one study including patients above the age of 8 and below the age of 23). A summary of PROMs and the parameters utilized are displayed in Table 2. Although both PROMs and clinical outcomes will be discussed in context for each section below, a separate section focused on the growing importance of PROM in predicting clinical outcomes follows a more general review of the PROMs.

### 3.2. Global Health Status Measurement/General Health Measurement

“Global health status” or “general health measurement” is a multidimensional construct encompassing physical, social, and emotional well-being, and has been popularized as an overall proxy of the patient’s QOL. Significant improvements in global health status are a key metric in clinical trials, given the relevance to the patient’s overall health status [19]. However, one of the challenges in applying the global health status to the setting of acute and chronic leukemias is the overall heterogeneity of disease presentation in both acute and chronic leukemias, even within the same subset [23]. Disease-specific PROM may help to offset the heterogeneity of complex diseases such as leukemia and MDS. Examples include the Life Ingredient Profile for Hematologic Malignancies, the Functional Assessment of Cancer Therapy– Leukemia (FACT-Leu) Questionnaire and the Medical Research Council/EORTC Quality of Life Questionnaire-Leukemia Module, a measure of HRQOL for patients with leukemia in remission. Additional models currently undergoing validity testing include the EORTC Quality of Life Module for Chronic Lymphocytic Leukemia and the Module for Chronic Myeloid Leukemia [3,10]. To date, relatively fewer clinical trials in acute or chronic leukemias or MDS include studies of HRQOL compared to those of their solid tumor counterparts [3,22,24].

In general, trials examining HRQOL in hematologic malignancies utilize patient-reported outcome measures (PROMs) such as the EORTC QLQ-30 and the FACT-General (FACT-G) [4,8,10]. Although the global health status has been devised by the EORTC QLQ-C30 and other common PRO measures as an overall, quantifiable perception of the QOL, they have been aptly modeled to balance extrinsic personal and social factors that may contribute to the ultimate determination of the patient’s responses. For example, for individuals without adequate health insurance coverage undergoing induction therapy for leukemias such as AML, the overall costs of care may be overwhelming, and such financial difficulties may accentuate the magnitude of the socioemotional aspects of the patient’s care, which is one factor captured in the global health status score [11]. Indeed, the concept of financial toxicity has received more attention across the spectrum of cancer care among patients with solid malignancies but has been relatively understudied among patients with hematologic malignancies [25].

### 3.3. Patient Symptoms

The presence and magnitude of symptoms vary among patients and over time for the same patient, and recently, the incorporation of PRO in many clinical trials has allowed further exploration into these heterogeneous presentations. Such variability makes it essential on both an individual and population level to capture this wide spectrum through longitudinal patient assessments. Symptoms are embedded in many PRO measures, such as those developed by the EORTC but are also available as stand-alone measures, such as the National Comprehensive Cancer Network (NCCN) disease-focused symptom indexes [19]. The core questionnaires (including the Functional Assessment of Cancer Therapy (FACT) underline the basis of the modular approach to QOL assessment adopted by the EORTC-QLG and are complemented by a range of available modules. This modular approach strives to improve the sensitivity and specificity of assessments of QOL in specific patient cohorts and allows the patient experience to be assessed so that both differences between groups and changes over time can be captured.

In our literature review, we found that common symptoms reported for patients with leukemias and MDS are fatigue, nausea, vomiting, pain, dyspnea, insomnia, and appetite loss. Fatigue, the most ubiquitous symptom reported in cancer patients overall, is particularly pronounced in acute and chronic leukemias and MDS compared to solid tumors [8,11,13]. For example, in one randomized control trial, clinical improvement in fatigue was documented at regularly scheduled visits over a 27-month observation period in the intervention arm of CLL patients receiving first-line chemoimmunotherapy with obinutuzumab plus bendamustine [11]. Of note, the EORTC QLQ-C30 was the most common PRO measure used among the clinical studies covered in this review, and symptoms covered in the survey include three symptom scales for fatigue, pain, and nausea/vomiting, but lack other common symptoms frequently reported by patients with hematologic malignancies, such as chills, fever, pruritis, and confusion [10,11,12,13,14,24]. Beyond the omission of several hematology-specific symptoms in the EORTC QLQ-C30, a review of included studies revealed further gaps in content coverage across PRO instruments. Domains such as body image, fear of recurrence, financial toxicity, and return to work were infrequently assessed despite their known relevance to survivorship in hematologic malignancies. Measures focusing on emotional well-being (e.g., depressive symptoms, anxiety, and uncertainty) were variably included and often captured through single-item scales, limiting interpretive depth. Furthermore, the timing of PRO assessments was highly heterogeneous; in several trials, PROs were collected only at baseline or end of treatment, which restricts understanding of symptom trajectories and recovery patterns over time. Reporting on missing data was inconsistent, with few studies describing the proportion or handling of incomplete PRO responses. Adjustment for key clinical or demographic confounders—such as disease risk category, treatment intensity, or comorbidities—was rarely detailed, potentially influencing observed PRO differences between subgroups [10,11,12,14,23,26,27,28]. Given the fluctuating, heterogenous and often multi-faceted nature of symptoms in cancer care, it may be difficult to actively encompass and distinguish symptoms of routine cancer care from symptoms of the underlying disease [23]. The greater use of electronic PROM shows promise in terms of providing increasingly accurate longitudinal measures for symptoms in patients with complex diseases such as hematologic malignancies [14,23].

### 3.4. Emotional Function

Diagnosis and treatment of cancer in general may be very stressful to patients, regardless of tumor type or stage, and exert an important emotional burden that may augment the adverse physical ramifications of the underlying disease [22]. In this regard, previous studies have documented that symptoms of clinically significant levels of depression are associated with poorer survival in cancer patients [22]. Furthermore, symptoms such as anxiety and depression may have a detrimental effect on a patient’s QOL. Interestingly, just as the diagnosis of cancer may beget negative emotional symptoms and subsequently predispose the patient to a mood disorder, there is also evidence that the patient’s neurobiology may interact with one’s immunology [12,22]. Several studies noted improvements in symptoms and QOL resulting from treatment in association with significant improvement in the emotional or cognitive functional scale after long-term follow-up [10,11]. Longitudinal monitoring should be emphasized to wholly capture the holistic, emotional experiences of patients, given that these scores have multiple determinants [23].

### 3.5. Physical Function

In exploring the recent PRO landscape, it is important to note that The EORTC, Short Form 36 Health Survey Questionnaire (SF-36), and FACT-G, include items related to objective measures of physical functionality, which can serve as a key endpoint in clinical management, similar to other physical metrics such as the Karnofsky functional scale, and may be important in connecting the realm of HRQOL and clinical outcomes [19]. Statistical models may be used to compare physical functional scores among diverse PRO surveys but may often not account for more granular characteristics such as gender, racial or other socioeconomic factors, which may impact physical functioning, which is also often overlooked in other PRO measures [1,26].

Self-reported physical well-being scores in multiple studies have been associated with OS and, as such, may have practical applications given the ability to correlate these data with treatment decisions for individuals with multiple comorbidities or limited physical mobility [4]. However, reported results of physical functional status vary greatly according to type and length of treatment across the diverse studies, which leads to heterogeneous results and difficulty in data interpretation and correlation with clinical outcomes [20]. This heterogeneity in response to treatment provides a plausible explanation for the fact that only a few of the studies included in this review showed significant differences in physical functioning [8,10,13].

### 3.6. Social Role/Function

PROs are essential markers of health and wellness when externally observable patient-important outcomes are often rare or unavailable. Namely, PROs provide a reasonable strategy for evaluating the treatment impact of complex diseases such as cancer, where patients may suffer from chronic pain, nausea, fatigue, sexual dysfunction, and emotional effects such as anxiety, for which objective physiologic measures may be limited in scope. Social function extends the umbrella of health and wellness past traditional physical and emotional markers of modern society [23]. Role function describes a patient’s structural position in the community or household, for example, spouse, worker, or parent. In contrast to role function, social function is multifaceted and is based on an individual’s membership in certain social groups (for example: being a Democrat, Hispanic, or Christian). Although social function is often assessed together with role function, in various instruments, these measures are often reported distinctly. In addition, unlike the other PROs discussed above, measurement of social and role functions can be more subjective and open to interpretation than other measures such as physical functioning [19].

Oftentimes, there are strong ties between role and social function. The complex relationship between role function, social function and HRQOL can be further highlighted by one study, which emphasized that higher social capital is strongly associated with better subjective health and higher lifestyle satisfaction overall [27]. As patients at higher levels of role function typically fill societal, community and family roles with positions of responsibility, this metric becomes fundamental in understanding how clinical treatment may holistically improve a patient’s HRQOL after intervention. Given this context, it was notable that improvement in social function was seen after treatment, particularly in studies with meaningful improvement in symptoms (namely, overall fatigue) [8,11,13]. In one study of patients with acute promyelocytic leukemia, marked improvement in role functioning was noted in the treatment group of patients who received arsenic trioxide therapy, which was the resultantly highest magnitude of change when compared to any of the other measured functional scales or global health status [13].

### 3.7. Relationships Between PRO Measures and Clinical Outcomes

Ultimately, the goal of providing healthcare, regardless of the modern sociopolitical landscape, is to restore or preserve physical and emotional function in a setting of overall health and wellness. This can be a daunting challenge in oncologic practice, given that many distressing symptoms are multifactorial and both objective and subjective in nature [28,29]. In these complex clinical settings, the use of PROs to systematically characterize a patient’s experiences may provide valuable information for the clinician to assist in clinical decision-making, particularly for challenging conditions such as hematologic malignancies. PROs are associated with clinical outcomes such as DFS and OS, particularly in older adults. Indeed, FACT-Leu scales, HRQOL and physical functioning scores were independent prognosticators of OS in geriatric patients with AML, underlining the growing significance of PRO in both clinical and research settings [4,12,19,23,26,30]. Nonetheless, the degree of this association and its clinical application should be the subject of further independent study, and it is of paramount importance to verify these results with future studies. One recent study by Efficace et al. suggests that, compared to baseline and at the 2-month mark, the risk of HRQoL deterioration was lower in the DEC arm than in the 3 + 7 arm (although non-significant at longer-term evaluation points at 6 and 12 months) and that lower-intensity treatment with DEC may be preferable to standard treatment in fit, older patients, indicating how the use of PROs may reliably influence clinical oncologic practice [30]. Notably, it may be gleaned that DEC was superior with respect to OS and DFS, whereas 3 + 7 was superior for HRQoL.

## 4. Discussion

Our literature review indicates that there are diverse PROMs designed to measure global status, symptoms, physical, emotional and social/role functions. We utilized a multipronged approach to identify and review recent literature that provided both PRO and clinical outcomes data, as well as a narrative overview of frequently used PROMs. Notably, analysis of several clinical studies of MDS and both acute and chronic leukemias provided the opportunity to outline unifying themes related to the PRO landscape of each disease that may be unique to the hematologic malignancies studied in this review. Ultimately, the goal was to identify trends and issues by examining both generic and disease-focused PROs. Beyond statistical associations, the clinical interpretability of changes in PRO scores is critical, and where available, minimal clinically important differences provide a more meaningful framework for understanding whether observed changes reflect patient-perceived benefit or harm.

The measurement and study of PROs is an evolving field, and provides ample room for exploring the important relationships between morbidity, mortality and the subjective patient experience [4,5]. The addition of PROMs such as the Fact-Leu, or Specific Indexes Physical Well-being score to prognostic scoring systems in AML and other hematologic malignancies may help identify patients at high-risk for disease- or treatment-related morbidity, although further research would be necessary to better understand the scope of PRO in clinical practice [4,6,8,9,10,14,15,16,17,18,31].

Our research had some limitations. First, although an attempt was made to convert this study into a systematic literature review or meta-analysis to provide better statistical analysis and scientific evidence, this was ultimately not feasible due to the heterogeneity of the studies and the paucity of data thus far available for PRO in hematologic malignancies, particularly in the context of clinical outcomes. Secondly, although chronic leukemia patients (CML/CLL) typically have better clinical outcomes than those with acute leukemia and MDS, this heterogeneous population is included in the literature review to provide a more inclusive, generalized overview of the milieu of PRO in leukemic diseases. In addition, a significant amount of data used for this review was derived from randomized controlled trials (RCTs) and/or clinical trials, each with specific eligibility criteria, treatment interventions, and outcome assessments, which may limit the generalizability of our results. Although an effort was made to include studies that assessed both adults and pediatrics for a more comprehensive review, there were very few studies with multiple participants younger than the age of 18, which limits any generalizability of our findings in the pediatric age group. In addition, some potential studies meeting our criteria may have been missed by the search strategy if there was no mention of the use or association of PROMs, PRO or HRQOL in the study, despite possible reporting of a patient’s subjective experiences or physical/emotional symptoms. Observed associations between PROs and clinical outcomes may be confounded by treatment intensity and toxicity profiles, as more intensive regimens and higher adverse-event burdens can independently worsen patient-reported symptoms and quality of life, irrespective of antitumor efficacy.

Overall, the current gap in the literature makes it challenging to provide a robust, overarching context of any one PRO, and particularly so for the general health measurement, given its multifaceted nature.

Investigating differences in PRO in various ethnic minorities and socioeconomic groups may further highlight areas in which we may conduct research that may help delineate gaps in the current knowledge for vulnerable populations. In addition, further development of disease-specific PRO instruments tailored to leukemias and MDS, given gaps in current measures such as financial toxicity, as well as improved timing and frequency of PRO assessments in clinical trials to better capture the trajectory of symptom burden and quality of life across treatment and survivorship, may greatly improve clinical adaptability of PRO. Finally, further use of PROs in novel trial designs, such as pragmatic or adaptive trials, to evaluate both efficacy and patient-centered outcomes in diverse populations may be another area of future research and development.

Still, the use of PRO surveys stands to increase communication between patients and oncologic healthcare providers and thereby facilitate healthy dialog that may be used to fuel constructive changes to the healthcare environment [4]. Given the complex relationship cancer patients may experience with the healthcare system, providing a safe haven for their voices to potentially impact oncologic care is a powerful tool for present and future clinicians and policymakers alike [23]. The absence of standardized PRO assessment timepoints across trials further limits cross-study comparability and synthesis, highlighting the need for greater harmonization of PRO collection schedules in future interventional studies.

## 5. Conclusions

The growing international recognition of the patient’s perspective in ensuring equitable, compassionate, and outcome-driven care cannot be overstated, and may be best achieved through identifying ways in which practitioners may better utilize PROMs in clinical research and practice. Through our review, notable trends were identified, which further highlight the practical importance of greater incorporation of PROMs in future clinical trials, particularly in the understudied realm of hematologic malignancies, in order to better delineate the link between survival and HRQOL [32,33].

In addition, important gaps in the current landscape of PROMs in hematologic malignancies remain. Additional studies should seek to identify the correlation between PRO and types of services received (such as oncologic care, palliative care, or other specialist consultation) as well as the setting in which they were administered (i.e., academic vs. community hospitals). In addition, clinical studies should seek longer-term follow-up (over 5–10 plus years) of HRQOL measures after the completion of treatment to further understand the association and potential impact of cancer survivorship on PRO. In a similar vein, the need for broader and more standardized incorporation of PROM in future interventional trials is of the utmost importance, particularly in the understudied realm of hematologic malignancies. Finally, as international clinical research collaboration increases, so does the need for cross-culturally relevant patient outcome assessment tools to embed proper semantic equivalence with culturally relevant assessment screening.

## Figures and Tables

**Table 1:** Literature review of clinical trials and patient-reported outcomes in myeloid diseases and leukemias.

Study Name/Number	Purpose of the Study	PRO Measure	Primary Clinical Outcome(s)	Disease	Age/Sample Size	Authors/Year of Publication	Conclusions
Patient-reported outcomes predict overall survival in older patients with acute myeloid leukemia [4] (CR107273)	The authors examined whether the Functional Assessment of Cancer Therapy—Leukemia (FACT-Leu) predicted OS beyond established prognostic factors among older patient with AML	FACT-Leu Physical Well-Being (PWB), Trial Outcomes Index (TOI), and Total scales	OS (overall survival)	AML	Median age approximately 60 years of age, *n* = 326	Peipert et al., 2021	These results indicate PROs’ value for predicting outcomes among older AML patients and underscore the need to more systematically collect PRO data in routine care with these patients
Patient-reported outcomes from a phase 3 randomized controlled trial of inotuzumab ozogamicin versus standard therapy for relapsed/refractory acute lymphoblastic leukemia [8] (NCT01564784)	The authors reported the impact of treatment with InO or SOC chemotherapy on PROs from the INO-VATE trial in adults with relapsed/refractory B-cell ALL	EORTC QLQ-C30 and the EuroQoL 5 Dimensions Questionnaires	OS, PCR (pathologic complete response)	ALL	Median age 46.5 years of age, *n* = 326	Kantarjian et al., 2018	The current PRO data support the favorable benefit/risk ratio of InO for the treatment of relapsed/refractory acute lymphoblastic leukemia, with superior clinical efficacy and better QoL
Patient-reported quality of life after tisagenlecleucel infusion in children and young adults with relapsed or refractory B-cell acute lymphoblastic leukemia: a global, single-arm, phase 2 trial [9]	The authors aimed to evaluate patient-reported quality of life in these patients before and after tisagenlecleucel infusion	Pediatric Quality of Life Inventory (PedsQL) and European Quality of Life-5 Dimensions questionnaire (EQ-5D)	CR, EFS (event-free survival), OS	ALL	Age 8–23 years, *n* = 107	Laetsch et al., 2019	Findings suggest a favorable benefit–risk profile of tisagenlecleucel in the treatment of pediatric and young adult patients with relapsed or refractory B-cell acute lymphoblastic leukemia
(NCT02435849) Health-related quality of life and patient-reported outcomes of ofatumumab plus chlorambucil versus chlorambucil monotherapy in the COMPLEMENT 1 trial of patients with previously untreated CLL [3] (NCT00748189)	Report patient-reported outcomes (PROs), including HRQoL parameters and patient-reported symptoms, from the COMPLEMENT 1 study.	EORTC QLQ-C30 version 3 and the EORTC QLQ-CLL16	DFS (disease-free survival)	CLL	Adults (ranging 35–92 years), *n* = 447	Hillmen et al., 2016	Data on health-related quality of life suggest that adding ofatumumab to patients’ treatment did not have negative impacts; it may have actually improved certain aspects of well-being
Patient-reported outcomes in the phase 3 BEFORE trial of bosutinib versus imatinib for newly diagnosed chronic phase chronic myeloid leukemia [10] (NCT02130557)	To utilize PRO instruments “for the identification of optimal CML therapy for individual patients” because of the chronic symptom burden treatment poses. The purpose of the study is to improve HRQoL and reduce non-adherence to treatment, and also see if improvements in clinical outcomes with individual TKIs are compromised by deterioration in HRQoL	FACT-Leu and EuroQoL-5 Dimensions (EQ-5D) questionnaires	EFS, OS	Chronic phase CML	Patient age ≥18 years, *n* = 530	Cortes JE et al., 2019	The favorable results of PRO analysis (stable or improved HRQoL and functional health status) can be combined with primary results of improved clinical efficacy with bosutinib versus imatinib and “a manageable safety profile for bosutinib in patients with newly diagnosed CP CML”
Improvements in Health-Related Quality of Life and Symptoms in Patients with Previously Untreated Chronic Lymphocytic Leukemia: Final Results from the Phase II GIBB study of the combination of obinutuzumab and bendamustine [11] (NCT02320487)	To determine how “initiating treatment with chemoimmunotherapy (bendamustine/BG) or targeted therapies affects PROs in CLL” and how the HRQoL of patients relates to disease-related symptoms	EORTC QLQ-C30) 20 and the EORTC QLQ Chronic Lymphocytic Leukemia 16 (QLQ-CLL16) instrument	OS	(previously untreated) CLL	Median age 61 years old, *n* = 102	Danilov et al., 2021	Findings indicate that “patients receiving first-line chemoimmunotherapy with BG experience consistent improvements in HRQoL that could be sustained for 3 years after start of treatment.” Improvements were “noted in context of high tumor response rates”
Low-dose decitabine versus best supportive care in elderly patients with intermediate- or high-risk myelodysplastic syndrome (MDS) ineligible for intensive chemotherapy: final results of the randomized phase III study of the European Organization for Research and Treatment of Cancer Leukemia Group and the German MDS Study Group [12] (NCT00043134)	This is a randomized phase III trial comparing decitabine with sole BSC in patients with intermediate-/high-risk MDS ≥ 60 years old who are ineligible for intensive treatment”—quality of life as a secondary objective	EORTC Quality of Life Questionnaire C30 (version 3.0)	OS, PFS (progression-free survival)	MDS or CML	Patient age ≥ 60 years, *n* = 220	Lubbert et al., 2010	Quality of life analysis through PRO questionnaire revealed a trend in favor of the decitabine arm
Long-term quality of life of patients with acute promyelocytic leukemia treated with arsenic trioxide vs. chemotherapy [13] (NCT03096496)	This study performed a follow-up study to assess long-term HRQoL and late effects in patients previously enrolled in the APL0406 trial to understand the benefits and risks of arsenic trioxide (ATO) therapy in APL patients.	EORTC QLQ-C30, and the Short Form Health Survey 36 (SF-36)	OS	APL	Age: 18–71 years, *n* = 161	Efficace et al., 2021	Overall findings suggest that the greater and more sustained antileukemic efficacy of ATRA-ATO is also associated with better long-term patient-reported outcomes than ATRA chemotherapy
Health-related quality of life with fixed-duration venetoclax-obinutuzumab for previously untreated chronic lymphocytic leukemia: Results from the randomized, phase 3 CLL14 trial [6] (NCT02242942)	The authors tested the treatment efficacy [of Ven-Obi combination drugs versus Cib-Obi] in conjunction with the patients’ health-related quality of life (HRQoL), and further evaluated the effects of treatment on symptoms as well as functional/HRQoL burden associated with both disease and treatment within this patient population	MDASI core instrument and CLL module and the EORTC QLQ-C30	DFS	CLL	Median age: 72 years, *n* = 445	Al-Sawaf et al., 2021	This analysis demonstrates that higher efficacy of Ven-Obi is not associated with QoL impairment and that Ven-Obi achieves early relief of CLL-related symptoms in elderly unfit patients
High response rate and improved exercise capacity and quality of life with a new regimen of darbepoetin alfa with or without filgrastim in lower-risk myelodysplastic syndromes: a phase II study by the GFM [14] (NCT00443339)	The authors tested in this trial the efficacy of a modified dosage of darbepoetin alfa of 500 μg every 2 weeks with or without G-CSF with efficacy being evaluated in terms of erythroid response, quality of life, and exercise function	SF-36 and FACT-An (for anemia) tests	OS	MDS	Median age: 72 years, *n* = 99	Kelaidi et al., 2013	Darbepoetin 500 μg every 2 weeks ±G-CSF was an effective and safe induction regimen for anemia in lower-risk MDS, associated with favorable long-term clinical outcomes in responders, including patient-reported quality of life and objectively measured exercise capacity
Outcomes of switching to dasatinib after imatinib-related low-grade adverse events in patients with chronic myeloid leukemia in chronic phase: the DASPERSE study [15] (NCT01660906)	The objective of this study was to evaluate whether after switching to dasatinib these patients had resolution of their imatinib-related toxicities while maintaining or improving their clinical response and the patient-reported symptom burden as a secondary endpoint	MDASI-CML and quality of life on the EORTC QLQ-C30) questionnaire	DFS	Chronic phase CML	Median age: 57 years, *n* = 39	Kim et al., 2018	Overall, efficacy and quality of life/symptom burden improved in many patients, despite the onset of dasatinib-related adverse events in most patients. This suggests that imatinib-treated patients with chronic, low-grade adverse events could benefit from switching to treatment with dasatinib
Health-related quality of life of newly diagnosed chronic myeloid leukemia patients treated with first-line dasatinib versus imatinib therapy [1] (NCT02348957)	We performed a multicenter study to compare HRQOL of newly diagnosed CML patients treated with front-line dasatinib (cases) or imatinib (controls). The secondary objectives were: to describe patient-reported symptom prevalence between treatment groups and examine HRQOL differences by age groups	EORTC QLQ-C30 and the EORTC QLQ-CML24 (disease-specific) questionnaires	OS	Chronic phase CML	Mean age: 58 years, *n* = 223	Efficace et al., 2020	We found that CML patients treated with first-line dasatinib, who were able to reach at least a CCyR, report a significantly lower impact of therapy on their daily life compared to their peers treated with imatinib
Health-related quality-of-life in treatment-naive CLL/SLL patients treated with zanubrutinib versus bendamustine plus rituximab [16] (NCT03336333)	The current analysis compared the effects of zanubrutinib versus BR on patients’ health-related quality-of-life (HRQoL).	EORTC QLQ-C30 and EQ-5D-5L	PFS	CLL	Patient age ≥18 years, *n* = 590	Ghia et al., 2023	During the first 24 weeks of treatment noted in the study, zanubrutinib was associated with better HRQoL outcomes in patients with treatment-naive CLL/SLL without del(17p) compared to BR
The Effect of Lenalidomide on Health-Related Quality of Life in Patients With Lower-Risk Non-del(5q) Myelodysplastic Syndromes: Results From the MDS-005 Study [17] (NCT01029262)	Health-related quality of life (HRQoL), a predefined secondary end point, was assessed using the European Organization for Research and Treatment of Cancer Quality of Life Questionnaire-Core 30 questionnaire at baseline, week 12, week 24, every 12 weeks thereafter, and at discontinuation based on the phase III MDS-005 study	EORTC QLQ-C30	OS/DFS	MDS	Median age of 71 years old, *n* = 239	Santini et al., 2023	Lenalidomide did not adversely affect HRQoL in RBC-TD patients with lower-risk non-del(5q) MDS and response to lenalidomide was associated with significant improvements in HRQoL
Eltrombopag for Low-Risk Myelodysplastic SyndromesWith Thrombocytopenia: Interim Results of a Phase II, Randomized, Placebo-Controlled Clinical Trial (EQOL-MDS) [18] (NCT02912208)	This multicenter trial presents the second part long-term efficacy and safety results of eltrombopag in patients with low-risk MDS and severe thrombocytopenia, as it relates to both clinical outcomes and HRQoL	EORTC QLQ-C30	OS/DFS	MDS	Patient age ≥18 years, *n* = 174	Oliva et al., 2023	Although no difference in the frequency of grade 1–2 adverse events (AEs) was observed, a higher proportion of eltrombopag patients experienced grade 3–4 AEs. AML evolution and/or disease progression occurred in 17% of eltrombopag and placebo patients with no difference in survival times

**Table 2:** A summary of PROMs and their target population (References noted in the table, denoted in brackets).

PRO Instrument/Total Scale and Items	Measure	Patient Population
European Organization for Research and Treatment of Cancer Quality of Life Questionnaire (EORTC QLQ-30) Scales: 15 total; 5 functional (Physical, Role, Emotional, Cognitive, Social) 3 symptom (Fatigue, Pain, Nausea/Vomiting) 1 Global Health Status/QoL; 6 single-item symptom scales; Items: 30	Global health status/quality of life, functional scales (physical, role, emotional, cognitive, and social), symptom scales (fatigue, nausea, vomiting, and pain), cancer-related symptom scales [1,2,5] (dyspnea, sleep disturbance, appetite loss, constipation, diarrhea), financial status	Adults, all cancer types, including Leukemia (i.e., AML, CLL, ALL, CML)
European Quality of Life-5 Dimensions Questionnaire (EQ-5D) Scales: 5 dimensions (Mobility, Self-Care, Usual Activities, Pain/Discomfort, Anxiety/Depression) Items: 5 core items + 1 optional VAS	Mobility, self-care, usual activities, pain/discomfort, and anxiety/depression; visual analog scale for self-rated health state [1,2]	Adults, all cancer types including Leukemia (i.e., AML, CLL, ALL, CML);
EORTC QLQ-C30 version 3 Scales: 15 total; 5 functional (Physical, Role, Emotional, Cognitive, Social) 3 symptom (Fatigue, Pain, Nausea/Vomiting) 1 Global Health Status/QoL; 6 single-item symptom scales; Items: 30	Global health status/quality of life, functional scales (physical, role, emotional, cognitive, and social, symptom scales, cancer-related symptom scales (dyspnea, sleep disturbance, appetite loss, constipation, diarrhea), financial status [1,3,5]	Adults, all cancers, including chronic/acute leukemia and lymphoma
EORTC Quality of Life Questionnaire Chronic Lymphocytic Leukemia 16-item module (QLQ-CLL16) Scales: 4 multi-item scales Fatigue, Emotional Concerns, Worries/Fears, Symptom Burden Items: 16	Fatigue, treatment side effects, symptom burden, infection, social activities, future health worries [1,2]	Adults, previously untreated CLL
EORTC Quality of Life Questionnaire Chemotherapy-Induced Peripheral Neuropathy 20 (QLQ-CIPN20) Scales: 3 Sensory, Motor, Autonomic Items: 20	Peripheral neuropathic side effects of chemotherapy; sensory, motor, automatic, and symptoms; functioning [1,3,22]	APL, CLL, chronic phase CML, AML, ALL, CLL, MDS, APL
EORTC QLQ-CML24 (disease-specific) Scales: 6 multi-item scales + single items Includes Impact on Daily Life, Symptom Burden, Worry/Mood, Body Image, Satisfaction with Care, Social Participation Items: 24	Symptom burden, impact on daily life, impact on worry/mood, body image problems, satisfaction with care, satisfaction with social life [1,2,7]	Adults, all cancers, including chronic/acute leukemia and lymphoma
FACT-Leu version 4 Scales: 5 Physical Well-Being (PWB) Social/Family Well-Being (SWB) Emotional Well-Being (EWB) Functional Well-Being (FWB) Leukemia-Specific Subscale (LeuS) Items: 44	General and leukemia-specific health-related quality of life; physical, social, emotional, functional well-being [1,2]	Adults, AML, chronic phase CML, ALL, CLL
FACT-Leu Physical Well-Being (PWB) Scales: 1 Items: 7	General and leukemia-specific health-related quality of life; physical, social, emotional, functional well-being − physical well-being (PWB) was reported here [1]	Ages 18 and older, Leukemia, all types (i.e., AML, CLL, ALL, CML)
FACT-Leu Trial Outcomes Index (TOI) Scales: 3 (PWB + FWB + LeuS) Items: 26	Sum of physical and functional well-being and leukemia subscale domain scores [1,2]	Ages 18 and older, Leukemia, all types (i.e., AML, CLL, ALL, CML)
FACT-Leu Total scales Scales: 5 (all FACT-Leu subscales combined) Items: 44	Sum of physical, functional, social/family, and emotional well-being domain scores [1,2,3]	Ages 18 and older, Leukemia, all types (i.e., AML, CLL, ALL, CML)
FACT-An (for anemia) Scales: 1 anemia-specific subscale Items: 13	Well-being (physical, social/family, emotional, functional), anemia subscale [1]	Adult patients with anemia
MD Anderson Symptom Inventory for CML (MDASI-CML) Scales: 3 (conceptual domains) Items: 26	Symptom (general and CML-specific) severity/interference [1,2]	Chronic phase CML
Pediatric Quality of Life Inventory (PedsQL) Scales: 4 (Physical, Emotional, Social, School Functioning) Items: 23	Health-related perceptions of quality of life: Emotional, social, and school functioning; physical and psychosocial health [1,2,3]	Ages 8–18, Chronic conditions in children, including ALL, AML, CLL, CML
Work Productivity and Activity Impairment (WPAI) Scales: 4 Items: 6	Work/activity impairment [2,7]	Adults, all cancers, including chronic/acute leukemia and lymphoma

## Data Availability

No new data were created or analyzed in this study. Data sharing is not applicable to this article.

## Appendix A. Search Strategy for Narrative Literature Review

Database Search Strategy:

A detailed search was subsequently conducted in MEDLINE and the Cochrane Library (used primarily for additional references in our narrative synthesis) for studies published between 2013 and 2025. The search was conducted in 2025 and was designed to identify studies related to patient-reported outcomes (PROs) in patients with myelodysplastic syndromes (MDS) and/or acute or chronic leukemias (AML, CML, ALL, or CLL), with a focus on survival outcomes such as overall survival (OS), disease-free survival (DFS), event-free survival (EFS), and progression-free survival (PFS).

Search Terms:

The following notable search terms were used in MEDLINE and the Cochrane Library:

1. Keywords:
  ○ “patient-reported outcomes”
  ○ “AML” (acute myeloid leukemia)
  ○ “CML” (chronic myeloid leukemia)
  ○ “ALL” (acute lymphoblastic leukemia)
  ○ “CLL” (chronic lymphocytic leukemia)
  ○ “MDS” (myelodysplastic syndromes)
2. Medical Subject Headings (MeSH):
  ○ “Leukemia, Myeloid”
  ○ “Leukemia, Lymphoid”
  ○ “Myelodysplastic Syndromes”
  ○ “Patient Reported Outcome Measures”
  ○ “Survival Rate”
  ○ “Disease-Free Survival”
  ○ “Progression-Free Survival”

Exclusion Criteria:

Studies were excluded based on the following criteria:

- Written in a language other than English.
- Not peer-reviewed publications.
- Conference abstracts or editorial papers.

Eligibility Criteria:

Studies were included in the review if they met the following criteria:

- Enrolled patients with MDS and/or AML, CML, ALL, or CLL
- Included PRO instruments or measures
- Reported survival outcomes (OS, DFS, EFS, PFS)

Additional Search Methods Utilized:

In addition to the primary database search, additional references were identified through:

- Backward citation searching: Hand-searching reference lists of included studies and systematic reviews.
- Forward citation searching: Using Google Scholar and the Cochrane Library to identify articles that cited the original included studies, which were ultimately included in the narrative synthesis, although were not listed as the 15 main studies for analysis.

Study Selection Process and Example Search:

A total of 1292 studies were identified initially with the following keywords “patient-reported outcomes”, “AML” (acute myeloid leukemia), “CML” (chronic myeloid leukemia), “ALL” (acute lymphoblastic leukemia), “CLL” (chronic lymphocytic leukemia), “MDS” (myelodysplastic syndromes) primarily in MEDLINE. After applying the filters for English full-length articles between the years 2013–2025 (and excluding papers featuring reviews, meta-analysis, editorials and non-clinical work), a total of 157 studies were found. Additional screening of the 157 studies were done using title and abstract screening to obtain 15 studies which were ultimately included in the review for detailed full-text analysis, which may be found in the primary manuscript document. Additional backward citation searching and forward citation searching was used in Cochrane Library for additional citations referred in the text.

## References

[1] Efficace F., Stagno F., Iurlo A., Breccia M., Cottone F., Bonifacio M., Abruzzese E., Castagnetti F., Caocci G., Crugnola M. (2020). Health-related quality of life of newly diagnosed chronic myeloid leukemia patients treated with first-line dasatinib versus imatinib therapy. Leukemia.

[2] Cocks K., Wells J.R., Johnson C., Schmidt H., Koller M., Oerlemans S., Velikova G., Pinto M., Tomaszewski K.A., Aaronson N.K. (2023). Content validity of the EORTC quality of life questionnaire QLQ-C30 for use in cancer. Eur. J. Cancer.

[3] Hillmen P., Janssens A., Babu K.G., Kloczko J., Grosicki S., Manson S., McKeown A., Gupta I., Chang C.-N., Offner F. (2016). Health-related quality of life and patient-reported outcomes of ofatumumab plus chlorambucil versus chlorambucil monotherapy in the COMPLEMENT 1 trial of patients with previously untreated CLL. Acta Oncol..

[4] Peipert J.D., Efficace F., Pierson R., Loefgren C., Cella D., He J. (2022). Patient-reported outcomes predict overall survival in older patients with acute myeloid leukemia. J. Geriatr. Oncol..

[5] Dawson J., Doll H., Fitzpatrick R., Jenkinson C., Carr A.J. (2010). The routine use of patient-reported outcome measures in healthcare settings. BMJ.

[6] Al-Sawaf O., Gentile B., Devine J., Zhang C., Sail K., Tandon M., Fink A., Kutsch N., Wendtner C., Eichhorst B. (2021). Health-related quality of life with fixed-duration venetoclax-obinutuzumab for previously untreated chronic lymphocytic leukemia: Results from the randomized phase 3 CLL14 trial. Am. J. Hematol..

[7] Ferrari R. (2015). Writing narrative style literature reviews. Med. Writ..

[8] Kantarjian H.M., Su Y., Jabbour E.J., Bhattacharyya H., Yan E., Cappelleri J.C., Marks D.I. (2018). Patient-reported outcomes from a phase 3 randomized controlled trial of inotuzumab ozogamicin versus standard therapy for relapsed or refractory acute lymphoblastic leukemia. Cancer.

[9] Laetsch T.W., Myers G.D., Baruchel A., Dietz A.C., Pulsipher M.A., Bittencourt H., Buechner J., De Moerloose B., Davis K.L., Nemecek E. (2019). Patient-reported quality of life after tisagenlecleucel infusion in children and young adults with relapsed or refractory B-cell acute lymphoblastic leukemia: A global single-arm phase 2 trial. Lancet Oncol..

[10] Cortes J.E., Gambacorti-Passerini C., Deininger M.W., Mauro M.J., Chuah C., Kim D.-W., Milojkovic D., le Coutre P., Garcia-Gutierrez V., BFORE Study Investigators (2019). Patient-reported outcomes in the phase 3 BFORE trial of bosutinib versus imatinib for newly diagnosed chronic phase chronic myeloid leukemia. J. Cancer Res. Clin. Oncol..

[11] Danilov A.V., Yimer H.A., Boxer M.A., Burke J.M., Babu S., Li J., Mun Y., Trask P.C., Masaquel A.S., Sharman J.P. (2022). Improvements in health-related quality of life and symptoms in patients with previously untreated chronic lymphocytic leukemia: Final results from the phase II GIBB study of obinutuzumab and bendamustine. Clin. Lymphoma Myeloma Leuk..

[12] Lübbert M., Suciu S., Baila L., Rüter B.H., Platzbecker U., Giagounidis A., Selleslag D., Labar B., Germing U., Salih H.R. (2011). Low-dose decitabine versus best supportive care in elderly patients with intermediate- or high-risk myelodysplastic syndrome ineligible for intensive chemotherapy: Final results of a randomized phase III study. J. Clin. Oncol..

[13] Efficace F., Platzbecker U., Breccia M., Cottone F., Carluccio P., Salutari P., Di Bona E., Borlenghi E., Autore F., Levato L. (2021). Long-term quality of life of patients with acute promyelocytic leukemia treated with arsenic trioxide versus chemotherapy. Blood Adv..

[14] Kelaidi C., Beyne-Rauzy O., Braun T., Sapena R., Cougoul P., Adès L., Pillard F., Lambert C., Charniot J.C., Guerci A. (2013). High response rate and improved exercise capacity and quality of life with darbepoetin alfa with or without filgrastim in lower-risk myelodysplastic syndromes: A phase II study by the GFM. Ann. Hematol..

[15] Kim D.W., Saussele S., Williams L.A., Mohamed H., Rong Y., Zyczynski T., Pinilla-Ibarz J., Abruzzese E. (2018). Outcomes of switching to dasatinib after imatinib-related low-grade adverse events in patients with chronic myeloid leukemia in chronic phase: The DASPERSE study. Ann. Hematol..

[16] Ghia P., Barnes G., Yang K., Tam C.S., Robak T., Brown J.R., Kahl B.S., Tian T., Szeto A., Paik J.C. (2023). Health-related quality of life in treatment-naive CLL/SLL patients treated with zanubrutinib versus bendamustine plus rituximab. Curr. Med. Res. Opin..

[17] Santini V., Almeida A., Giagounidis A., Platzbecker U., Buckstein R., Beach C., Guo S., Altincatal A., Wu C., Fenaux P. (2018). Effect of lenalidomide on health-related quality of life in patients with lower-risk non–del(5q) myelodysplastic syndromes: Results from the MDS-005 study. Clin. Lymphoma Myeloma Leuk..

[18] Oliva E.N., Riva M., Niscola P., Santini V., Breccia M., Giai V., Poloni A., Patriarca A., Crisà E., Capodanno I. (2023). Eltrombopag for low-risk myelodysplastic syndromes with thrombocytopenia: Interim results of a phase II randomized placebo-controlled clinical trial (EQOL-MDS). J. Clin. Oncol..

[19] Kerrigan K., Patel S.B., Haaland B., Ose D., Chalmers A.W., Haydell T., Meropol N.J., Akerley W. (2020). Prognostic significance of patient-reported outcomes in cancer. JCO Oncol. Pract..

[20] Basch E., Deal A.M., Kris M.G., Scher H.I., Hudis C.A., Sabbatini P., Rogak L., Bennett A.V., Dueck A.C., Atkinson T.M. (2016). Symptom monitoring with patient-reported outcomes during routine cancer treatment: A randomized controlled trial. J. Clin. Oncol..

[21] Murugappan M.N., King-Kallimanis B.L., Reaman G.H., Bhatnagar V., Horodniceanu E.G., Bouchkouj N., Kluetz P.G. (2022). Patient-reported outcomes in pediatric cancer registration trials: A US Food and Drug Administration perspective. J. Natl. Cancer Inst..

[22] Sotelo J.L., Musselman D., Nemeroff C. (2014). The biology of depression in cancer and the relationship between depression and cancer progression. Int. Rev. Psychiatry.

[23] LeBlanc T.W., Abernethy A.P. (2017). Patient-reported outcomes in cancer care—Hearing the patient voice at greater volume. Nat. Rev. Clin. Oncol..

[24] Basch E., Deal A.M., Dueck A.C., Scher H.I., Kris M.G., Hudis C., Schrag D. (2017). Overall survival results of a trial assessing patient-reported outcomes for symptom monitoring during routine cancer treatment. JAMA.

[25] Villalona S., Castillo B.S., Chavez Perez C., Ferreira A., Nivar I., Cisneros J., Guerra C.E. (2024). Interventions to mitigate financial toxicity in adult patients with cancer in the United States: A scoping review. Curr. Oncol..

[26] Rivera S.C., Kyte D.G., Aiyegbusi O.L., Slade A.L., McMullan C., Calvert M.J. (2019). The impact of patient-reported outcome data from clinical trials: A systematic review and critical analysis. Health Qual. Life Outcomes.

[27] Imbulana Arachchi J., Managi S. (2023). The role of social capital in subjective quality of life. Humanit. Soc. Sci. Commun..

[28] Walshe C., Ewing G., Griffiths J. (2012). Using observation as a data collection method to help understand patient and professional roles and actions in palliative care settings. Palliat. Med..

[29] Van der Kruk S.R., Butow P., Mesters I., Boyle T., Olver I., White K., Sabesan S., Zielinski R., Chan B.A., Spronk K. (2022). Psychosocial well-being and supportive care needs of cancer patients and survivors living in rural or regional areas: A systematic review from 2010 to 2021. Support. Care Cancer.

[30] Calvert M., Blazeby J., Altman D.G., Revicki D.A., Moher D., Brundage M.D., CONSORT PRO Group (2013). Reporting of patient-reported outcomes in randomized trials: The CONSORT PRO extension. JAMA.

[31] Isfort S., Manz K., Teichmann L.L., Crysandt M., Burchert A., Hochhaus A., Saussele S., Kiani A., Göthert J.R., Illmer T. (2023). Step-in dosing of bosutinib in patients with chronic phase chronic myeloid leukemia after second-generation tyrosine kinase inhibitor therapy: Results of the bosutinib dose optimization study. Ann. Hematol..

[32] Cannella L., Caocci G., Jacobs M., Vignetti M., Mandelli F., Efficace F. (2015). Health-related quality of life and symptom assessment in randomized controlled trials of patients with leukemia and myelodysplastic syndromes: What have we learned?. Crit. Rev. Oncol. Hematol..

[33] Gao P., Bai P., Kong X., Fang Y., Gao J., Wang J. (2022). Patient-reported outcomes and complications following breast reconstruction: Comparison between biological matrix-assisted direct-to-implant and latissimus dorsi flap. Front. Oncol..

